# Comparison of Different Electrocardiography with Vectorcardiography Transformations

**DOI:** 10.3390/s19143072

**Published:** 2019-07-11

**Authors:** Rene Jaros, Radek Martinek, Lukas Danys

**Affiliations:** Department of Cybernetics and Biomedical Engineering, Faculty of Electrical Engineering and Computer Science, VSB–Technical University of Ostrava, 17. listopadu 15, 708 33 Ostrava, Czech Republic

**Keywords:** electrocardiography, vectorcardiography, transformation, Frank’s leads, Kors transformation, dower transformation, quasi-orthogonal leads, least-squares fit method

## Abstract

This paper deals with transformations from electrocardiographic (ECG) to vectorcardiographic (VCG) leads. VCG provides better sensitivity, for example for the detection of myocardial infarction, ischemia, and hypertrophy. However, in clinical practice, measurement of VCG is not usually used because it requires additional electrodes placed on the patient’s body. Instead, mathematical transformations are used for deriving VCG from 12-leads ECG. In this work, Kors quasi-orthogonal transformation, inverse Dower transformation, Kors regression transformation, and linear regression-based transformations for deriving P wave (PLSV) and QRS complex (QLSV) are implemented and compared. These transformation methods were not yet compared before, so we have selected them for this paper. Transformation methods were compared for the data from the Physikalisch-Technische Bundesanstalt (PTB) database and their accuracy was evaluated using a mean squared error (MSE) and a correlation coefficient (R) between the derived and directly measured Frank’s leads. Based on the statistical analysis, Kors regression transformation was significantly more accurate for the derivation of the X and Y leads than the others. For the Z lead, there were no statistically significant differences in the medians between Kors regression transformation and the PLSV and QLSV methods. This paper thoroughly compared multiple VCG transformation methods to conventional VCG Frank’s orthogonal lead system, used in clinical practice.

## 1. Introduction

Electrocardiography (ECG) is currently the most widely used method of sensing electrical activity in the human heart. Vectorcardiography (VCG) is an investigation method that was previously compared to ECG several times and was evaluated as a useful investigation method [1,2,3]. It achieved a higher sensitivity for the detection of hypertrophy as well as ischaemic heart disease [4,5,6,7]. Three leads scanned in three mutually orthogonal axes (vertical, transversal, and sagittal) are used for VCG measurements, but this is not commonly used in clinical practice due to the more complex interpretation of the recordings. Mathematical transformations can be made between ECG and VCG leads. Previously, ECG transformations from VCG leads were used, but later only 12-lead ECG spread and VCG leads were derived from ECG leads instead of measurements. These transformations are based on transformation coefficients that have been defined by various authors [8,9,10]. For example, the authors used thoracic models to obtain the coefficients, or, based on the similarity of one of the ECG leads, they determined the orthogonal lead. Transformation methods have also been developed based on a regression approach for multiple recordings of simultaneously measured ECG and VCG.

VCG is a diagnostic and less specific method than the standard 12-lead ECG measurement [1]. This method can be measured using several different lead systems. In clinical practice, Frank’s orthogonal lead system is the most widely used. It is measured using three orthogonal leads described by Frank, which measure cardiac activity with the same sensitivity. The problem of needing to connect multiple electrodes to measure both ECG and VCG was solved by synthesis from the currently recorded ECG leads. Compared to ECG, VCG has been recognized as a very useful investigative clinical method for cardiac activity sensing [1,3]. In addition, it shows the balance of cardiac cycle forces at any moment and contains more information in comparison to ECG that may have diagnostic significance, such as the display of temporal or phase relationships between complexes. The importance of VCG is, for example, in the detection and localization of acute myocardial infarction, right ventricular hypertrophy, and Tawar arm blockade. Despite all the benefits of VCG, ECG is increasingly being used.

**Figure 1:**
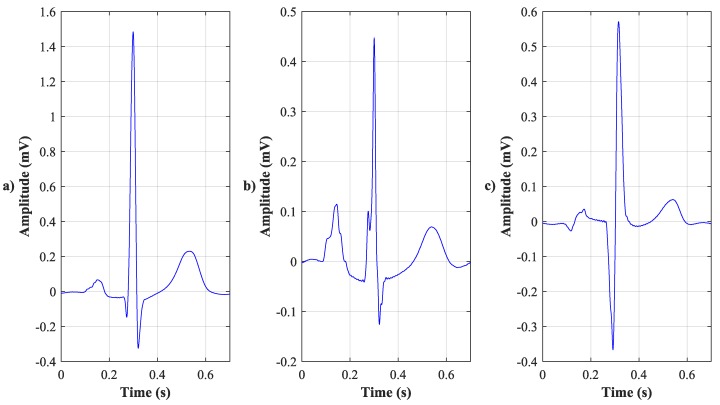
1-D image of three scalar vectorcardiographic (VCG) recordings. (**a**) X lead, (**b**) Y lead, and (**c**) Z lead.

**Figure 2:**
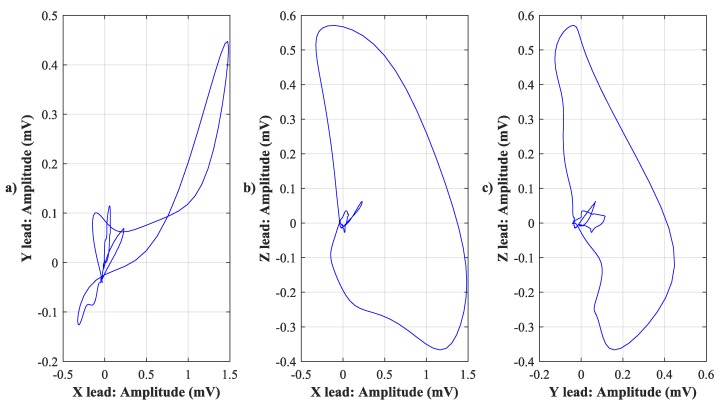
2-D image using three VCG planes. (**a**) X lead to Y lead, (**b**) X lead to Z lead, and (**c**) Y lead to Z lead.

**Figure 3:**
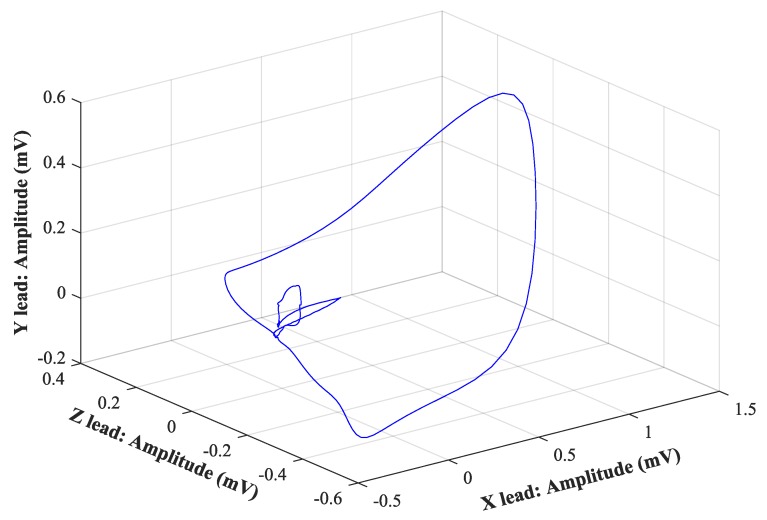
3-D image of VCG.

VCG recognizes three planes, sagittal, transversal, and frontal. The frontal one is located between the XY leads, the transverse one between the XZ leads, and the sagittal one between the YZ leads. The electrical activity of the heart is described by three loops that represent the individual phases, and their contours, rotation, or the direction of the cardiac axis are taken into account. The first loop corresponds to the P wave, the second one, which is also the largest loop, corresponds to the QRS complex, and the third one corresponds to the T wave. The loops can be displayed in a 1-D image as three scalar recordings (Figure 1), in a 2-D image using three planes (Figure 2), or in one 3-D image (Figure 3). The axes are represented as time and voltage dependence in the 1-D image. In the case of 2-D and 3-D images, the dependence between the individual leads in mV is displayed. In the analysis, the greatest attention was paid to the QRS complex, which is oval in shape and faces in the same direction as the cardiac axis of the heart [2].

**Figure 4:**
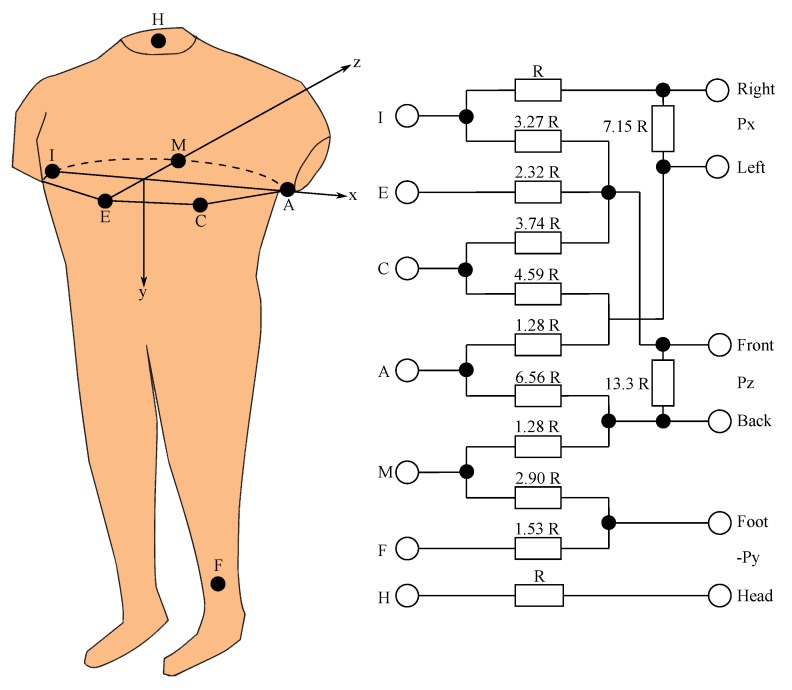
Frank’s orthogonal lead system.

The Frank’s orthogonal lead system is most widely used for its simplicity and is almost orthogonal. This is a bipolar circuit. It consists of seven electrodes whose position is marked with capital letters: I, E, C, A, M, F, and H.Each electrode has its specific location, see Figure 4. The E electrode is located on the front, in the middle of the chest, and the M electrode is located opposite, in the rear, on the back. The I electrode is located in the right central axillary line and the A electrode is in the left central axillary line. The position of the C electrode is obtained by halving the distance between the A electrode and E electrode. The F Electrode is positioned on the left leg and the H electrode on the neck. Furthermore, one electrode attached to the right leg is used as a ground electrode. On the X-axis, the left side of the body is positive with respect to the right side of the body, on the Y-axis, the left foot is a positive electrode with respect to the head, and on the Z-axis, the back of the chest is positive with respect to the front [2]. The individual electrodes are routed to a resistor network. Malmivuo et al., 1995 [11] explained the correct values of each resistors in their book. The signals on the leads are derived using mathematical Equations (1)–(3), where P represents the potentials on the individual electrode clips and I, E, C, A, M, F, H represent individual electrodes [8]. Macfarlane et al., 2010 [12] explained calculations of Equations (1)–(3), which are based on the resistor network in Figure 4. In addition to Frank’s orthogonal leads, other leads, such as McFee–Parungao leads [13] and Schmitt–Simonson [14] leads called Svec-III, are used for sensing VCG [15,16,17].

(1) $$P_{X}=0.610\cdot A+0.171\cdot C-0.781\cdot I.$$

(2) $$P_{Y}=0.655\cdot F+0.345\cdot M-1.000\cdot H.$$

(3) $$P_{Z}=0.133\cdot A+0.736\cdot M-0.264\cdot I-0.374\cdot E-0.231\cdot C.$$

There are many different transformation methods of 3-lead VCG to 8-lead ECG, or 3-lead ECG to 8-lead VCG. Kors et al., 1990 [18] compared the Kors quasi-orthogonal transform to the inverse Dower transform, the Kors regression transform, and the Frank’s orthogonal lead system. A total of 90 recordings were used. The regression method achieved the best results in this study, while the quasi-orthogonal method achieved the worst results. Other quasi-orthogonal transforms were described in articles [19,20,21,22]. The authors use different coefficients in equations to perform ECG to VCG transformations. Burger et al., 1952 [23] introduced first linear transformation method. They showed that it would be possible to transform ECG to VCG. Levkov, 1987 [3] introduced five different transformation methods in his work. One transformation method is based on one-dipole model and four transformation methods are derived by regression. His methods provide high accuracy, particularly th the methods based on regression. Edenbrandt et al., 1994 [4] tested the inverse Dower transformation. Authors compared this method with the directly measured Frank’s leads, the quasi-orthogonal method described by Bjerl, and the quasi-orthogonal method implemented by Marquette Electronics Inc. control systems. The comparison was performed on 80 people with different types of heart attacks. The inverse Dower transformation achieved the best QRS complex image with respect to other methods and its recording was the most similar to the Frank’s. Guillem et al., 2006 [9] tested linear regression-based transformations for deriving the P wave (PLSV) and QRS complex (QLSV). They compared these methods with the inverse Dower transformation methods during the QRS complex and P wave detection on 247 recordings. In the P wave transformation, the PLSV method achieved the best results and the QLSV method achieved the best results for QRS complex detection. Both methods achieved better results than the inverse Dower method. Acar and Koymen, 1999 [24] optimized the singular value decomposition (SVD) transformation to derive VCG indications. They used data from 23 patients. Their approach does not perform transformation of three VCG leads but provides three non-correlated orthogonal leads. Dawson et al., 2009 [25] used the Dower transform and statistical method called affine transform to derive 8-lead ECG from 3-lead VCG and their inverse forms to derive 3-lead VCG from 8-lead ECG. They concluded that for subjects with myocardial infarction and healthy control subjects, affine transform provides better accuracy than Dower transform. Maheshwari et al., 2016 [26] showed that principal component analysis (PCA) could also be used for deriving the VCG from ECG. They compared PCA with the inverse Dower transform and the Kors regression transform. Their results support the theoretical basis behind the PCA-based reconstruction methodology and show better performance then the compared transforms. Vozda et al., 2014 [27] used transformation methods based on artificial neural networks. They showed that these transformation methods offer even higher accuracy then other reviewed transforms.

In this paper, Kors quasi-orthogonal transform, inverse Dower transform, and Kors regression transform were chosen, because they are the most commonly used transformation methods. A very large number of transformation methods and matrices are optimizing the transformations of the QRS complex. Methods based on linear regression transformations are focused not only on the QRS complex, but also on optimizing the transformations of the P wave. That is the main reason why this transform was chosen.

## 2. Methods

The first transformations were designed to derive ECG from VCG leads. This was due to a reduction in the number of electrodes attached compared to the current ECG and VCG measurements. The first one was the Dower transformation, which was derived using Frank’s thoracic model and by calculating the linear coefficients [3]. Later, only 12-lead ECG spread in clinical practice while VCG ceased to be measured. Therefore, VCG leads were more often derived from ECG leads. 3 VCG leads X, Y, and Z are derived by transforming the 12-lead ECG. To transform the 12-lead ECG into 3 lead VCG, only the values of 6 thoracic leads, lead I and lead II, i.e., 8 linearly independent leads are used. The remaining 4 leads are linearly dependent and can therefore be calculated. At the same time, linearly dependent leads are not used because they could exacerbate the resulting transformation [3,8,9]. The mathematical transformation is based on the multiplication of matrices according to Equation (4), where V is a VCG matrix whose lines correspond to 3 VCG leads, M is a transformation matrix of the selected method, and E is a matrix whose lines are the individual ECG leads.

(4) V=M·E.

### 2.1. Kors Quasi-Orthogonal Transformation

This method is based on the assumption that the X lead is similar to the V6 lead to a certain extent, and the Y lead is similar to lead II. Z Lead partially corresponds to the similarity of the negative half of the V2 lead. Hence, all input leads have coefficients equal to zero, except for three selected leads similar to orthogonal leads. The resulting VCG leads can be obtained using Equations (5)–(7) [10,18].

(5) X=V6.

(6) Y=II.

(7) Z=−0.5·V2.

### 2.2. Inverse Dower Transformation

This method is based on the mathematical pseudoinversion of the Dower’s method of deriving ECG from VCG. Orthogonal leads X, Y, and Z are expressed by a linear combination of 8 leads. Transformation coefficients are represented in Table 1 [4,8,28,29].

### 2.3. Kors Regression Transformation

This is a statistical method for which Kors used mathematical regression [8,18]. The regression method is used when recordings from a large number of patients are available. Kors, while measuring ECG and VCG, derived mathematically the coefficients for the regression method, see Table 2. These coefficients allow ECG to be transformed into VCG. The reconstruction coefficients are obtained by minimizing the mean squared error (MSE) according to Equation (9) [8,18,29,30].

### 2.4. Linear Regression-Based Transformations

Least-square value (LSV) is based on a regression approach. By minimizing the MSE, the coefficients for two methods focused on another ECG interval were derived, see Table 3 and Table 4. Using the interval from the beginning to the end of the P wave, a matrix for transformation with emphasis on P wave (PLSV transform) was obtained. Using the interval from the beginning to the end of the QRS complex, a matrix for transformation with emphasis on the QRS complex (QLSV transform) was obtained [9,10].

## 3. Evaluation Parameters

The Physikalisch-Technische Bundesanstalt (PTB) [31,32,33] diagnostic database was used to test the ECG to VCG transformation. The database contains recordings measured by the Benjamin Franklin Cardiology Department, which were measured in healthy patients and patients with various heart diseases. Of the total of 289 entities, 549 recordings are recorded in the database. A total of 1 to 5 recordings were measured for each patient. This database has mixed recordings that consists of healthy entities and patients with pathological heart diseases. The individual recordings contain 15 simultaneously measured leads. The leads measured comprise of 12 ECG leads (Eithoven, Goldberg, and Wilson leads) and 3 VCG leads (Frank’s leads). The sampling frequency of each signal is 1 kHz. For the evaluation, 50 recordings of healthy patients were chosen from this database. This database is publicly accessible on Physionet [31,32,33].

The correlation coefficient (R) and the mean squared error (MSE) were chosen to test the accuracy of the transformation by the individual methods in relation to the reference, Frank’s leads. Subsequently, a statistical analysis using the Mann–Whitney nonparametric median test was selected.

### 3.1. Correlation Coefficient

Correlation indicates the relationship between two recordings or variables. It is often used in statistics, where a linear dependency between recordings is sought. The correlation coefficient does not have a unit and its value is in the interval of <−1,1>. If the correlation value is 0, there is no correlation between the two variables. If the value is −1, it means indirect linearity between the recordings. The value of a correlation equaling 1 then means direct linearity, i.e., similarity between the recordings evaluated. The equation for calculating the correlation coefficient is given by Equation (8), where *V* is the original value of the VCG measured, DV is the value of the VCG derived from the ECG, and *n* is the number of samples [34].

(8) $$R=\frac{\sum_{i=1}^{n}\left(V\cdot DV\right)}{\sqrt{\sum_{i=1}^{n}V^{2}\sum_{i=1}^{n}DV^{2}}}.$$

### 3.2. Mean Squared Error

The mean squared error compares two recordings based on similarities or differences. To evaluate the transformation’s accuracy, we compare MSE calculated between the transformed signal and the directly measured VCG in each lead. The VCG uses MSE to evaluate the amplitude error of the recording. The unit is the squared unit of the variable measured. The closer the result is to zero, the more similar or identical the recordings are [35]. The equation for calculating MSE is given by Equation (9), where *V* is the original value of the VCG measured, DV is the value of the VCG derived from the ECG, and *n* is the number of samples.

(9) $$MSE=\frac{1}{n}\sum_{i=1}^{n}{\left(V_{i}-DV_{i}\right)}^{2}.$$

### 3.3. Statistical Analysis

When testing the hypotheses, the significance level of α is determined. 95% is considered a reliable estimate and the significance level is the remaining 5% (0.05). If the difference between the two files tested is less than α, then the recordings are different at the significance level [36,37]. In statistics, the mean and the median are most often determined for performing a statistical test. Statistical analysis is based on hypotheses testing. The zero hypothesis (H${}_{0}$) represents the equilibrium (e.g., equality between the median results) and the alternative hypothesis (H${}_{A}$) then expresses the equilibrium violation (e.g., the difference between the median results). Based on the selected statistical test, either the zero hypothesis is rejected and the alternative hypothesis is accepted, or the zero hypothesis is not rejected and the alternative hypothesis is rejected. When an alternative hypothesis is reached between two compared transformations, the testing will be assessed by the significance level. For statistical analysis, we chose from statistical tests, which are divided into parametric and nonparametric ones. Parametric tests work with normal input data distribution and have a higher test power than nonparametric tests. The data used in this article do not originate from the normal distribution, therefore, the Mann–Whitney nonparametric median test is used [36,37].

## 4. Results

Before conducting individual transformations, signal pre-processing was performed using a frequency selective filter with a finite impulse response (FIR filter). Based on recommendations [38,39,40], filter cut-off frequencies should be selected from 0.05 to 100 Hz. Since we only analyzed the recordings of healthy volunteers, the limit values were set from 0.2 to 100 Hz with a filter order of 500, which is in line with the recommendation stated in the articles [38,39,40].

Subsequently, R-oscillations were detected using the Pan–Tompkins detector. The individual positions of the R-oscillations determine the beginning of the heart rate as R−300 ms and the end of the heart rate as R+400 ms. Thus, all heart rates of the individual leads were cut out. The resulting transformations could be influenced by extrasystoles, so they were removed using the library function in MATLAB. Once the extrasystoles were removed, the individual heart rates were averaged, so that the average heart rate for each ECG and VCG lead was created to perform the transformation and the transformation accuracy check.

The test was performed on the recordings from the PTB database and the MSE and R result vector in relation to the reference Frank’s leads was obtained for each method. Since the mean value of the results was different from the median of the results, it was determined that the data did not come from a normal distribution. Statistical testing of data normality was performed at work [27]. Therefore, the Mann–Whitney nonparametric median test was selected for statistical analysis. If the resulting *p*-value was less than the significance level of α=0.05, the zero hypothesis would be rejected in favor of the alternative hypothesis. This would mean that the difference between the medians compared was statistically significant. The determination of the zero and alternative hypotheses can be seen in Equations (10) and (11), where the zero hypothesis indicates that the median results were statistically identical, and the alternative hypothesis indicates that the difference between the median results was statistically significant. The test was bilateral, so the medians for Kors regression transformation and the method compared must be determined.

(10) $$H_{0}:{\tilde{x}}_{1}={\tilde{x}}_{2}.$$

(11) $$H_{A}:{\tilde{x}}_{1}\neq {\tilde{x}}_{2}.$$

### 4.1. Visual Evaluation

**Figure 5:**
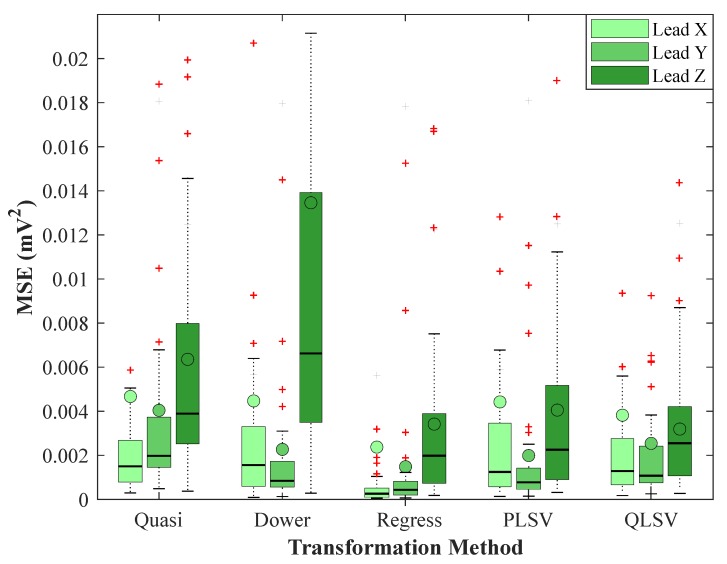
Box plot for comparison of the methods based on the mean squared error (MSE).

**Figure 6:**
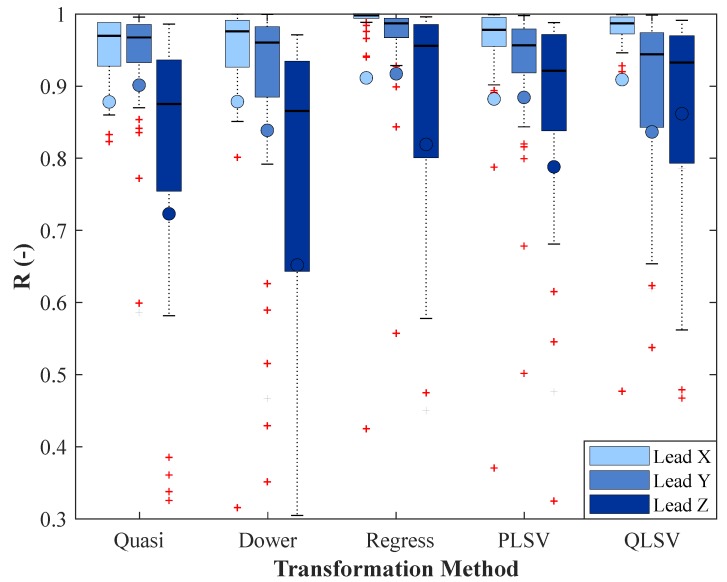
Box plot for comparison of the methods based on the correlation coefficient.

The testing focused on the regression method described by Kors because, according to studies, this transformation has achieved the best results in comparison to other transformations. Statistical analysis was performed for recordings measured on healthy volunteers that were received from the PTB database. For comparison, box plots are created, see Figure 5 and Figure 6. In the figures and tables, the result of the Kors quasi-orthogonal transformation will be referred to as *Quasi*, the result of inverse Dower transformation will be referred to as *Dower*, the result of Kors regression transformation will be referred to as *Regress*, the result of the PLSV method will be referred to as *PLSV*, and the result of the QLSV method will be referred to as *QLSV*. It can be concluded from the figure that Kors regression transformation was the most accurate because the median MSE was lower than the other methods for X and Y leads, see Figure 5. Similarly, it can be seen that the median R was higher than the other methods for the X and Y leads, see Figure 6. In the box plots, the median is represented by the horizontal line and the mean by the circle. Red crosses represent outliers, which are commonly used in statistics to represent samples which are removed from evaluation. Statistical verification that this method is better must be proved by a statistical test.

### 4.2. Statistical Analysis of MSE Results

Figure 5 indicates that the MSE medians of Kors regression transformation for X and Y leads are lower than the medians of the other transformation methods and, for Z lead, it could not be assessed. According to the statistical test, all *p*-values for the X and Y leads were less than the significance level of α=0.05, see Table 5, and, thus, it was impossible to reject the zero hypothesis. This means that Kors regression transformation has been recognized as more accurate for X and Y leads than all other transformation methods, based on the medians of the MSE results.

For Z lead, the *p*-values between Kors regression transformation and Kors quasi-orthogonal transformation, and between Kors regression transformation and inverse Dower transformation were less than the significance level of α=0.05, therefore, the zero hypothesis could not be rejected here. Thus, Kors regression transformation was recognized as more accurate for Z lead than Kors quasi-orthogonal transformation and inverse Dower transformation, based on the median of the MSE results. For Z lead, the *p*-values between Kors regression transformation and the PLSV method, and Kors regression transformation and the QLSV method were greater than the significance level of α=0.05, therefore, the zero hypothesis was rejected in favor of the alternative hypothesis. This indicates that, for Z lead, it cannot be asserted that Kors regression transformation would achieve a more accurate transformation than the PLSV and QLSV methods, hence, the difference between the methods was not statistically significant.

### 4.3. Statistical Analysis of R Results

Figure 6 indicates that the R medians of Kors regression transformation for X and Y leads were higher than the medians of the other transformation methods and, for Z lead, it could not be assessed with certainty. According to the statistical test, all *p*-values for the X and Y leads were higher than the significance level of α=0.05, see Table 6, and, thus, it was impossible to reject the zero hypothesis. This means that Kors regression transformation has been recognized as more accurate for X and Y leads than all other transformation methods, based on the medians of the R results. For Z lead, the *p*-values between Kors regression transformation and Kors quasi-orthogonal transformation, and between Kors regression transformation and inverse Dower transformation were less than the significance level of α=0.05, therefore, it was impossible to reject the zero hypothesis here, either. Thus, Kors regression transformation was recognized as more accurate for Z lead than Kors quasi-orthogonal transformation and inverse Dower transformation, based on the medians of the R results. For Z lead, the *p*-valuess between Kors regression transformation and the PLSV method, and Kors regression transformation and the QLSV method were greater than the significance level of α=0.05, therefore, the zero hypothesis was rejected in favor of the alternative hypothesis. This indicates that, for Z lead, it cannot be asserted that Kors regression transformation would achieve a more accurate transformation than the PLSV and QLSV methods based on the medians of the R results, hence, the difference between the methods was not statistically significant.

## 5. Discussion

**Figure 7:**
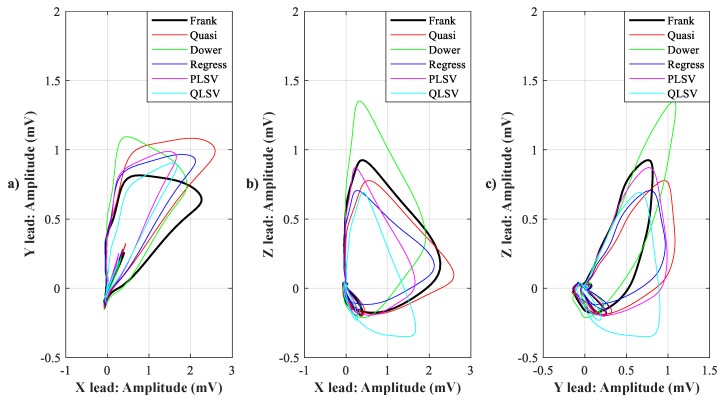
Comparison of transformation methods in 2-D, random patient 1. (**a**) X lead to Y lead, (**b**) X lead to Z lead, and (**c**) Y lead to Z lead.

**Figure 8:**
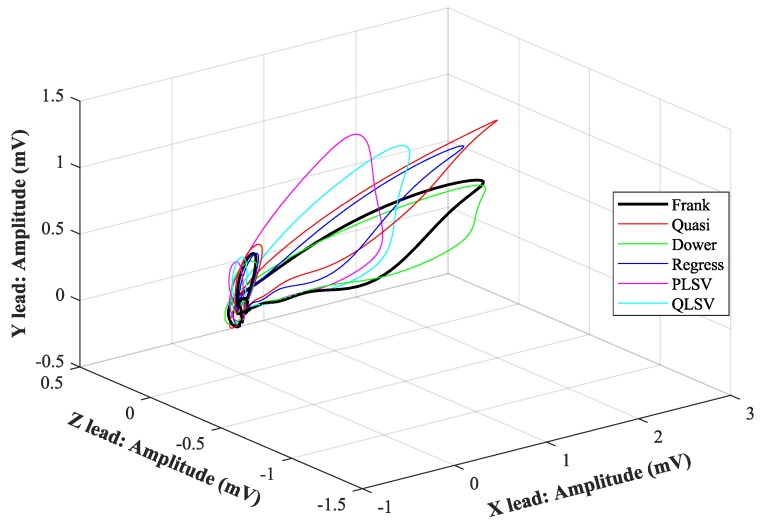
Comparison of transformation methods in 3-D, random patient 1.

**Figure 9:**
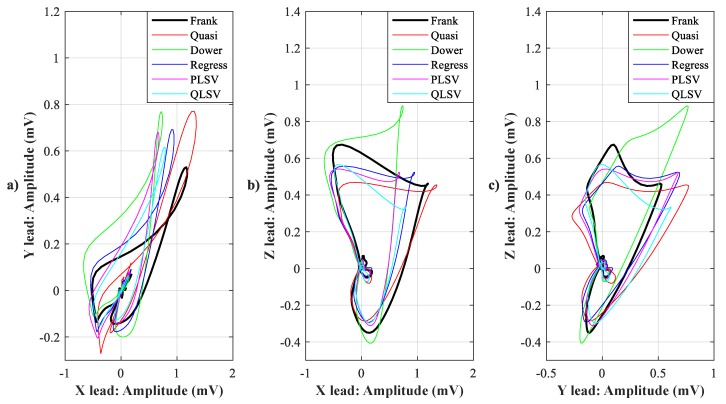
Comparison of transformation methods in 2-D, random patient 2. (**a**) X lead to Y lead, (**b**) X lead to Z lead, and (**c**) Y lead to Z lead.

**Figure 10:**
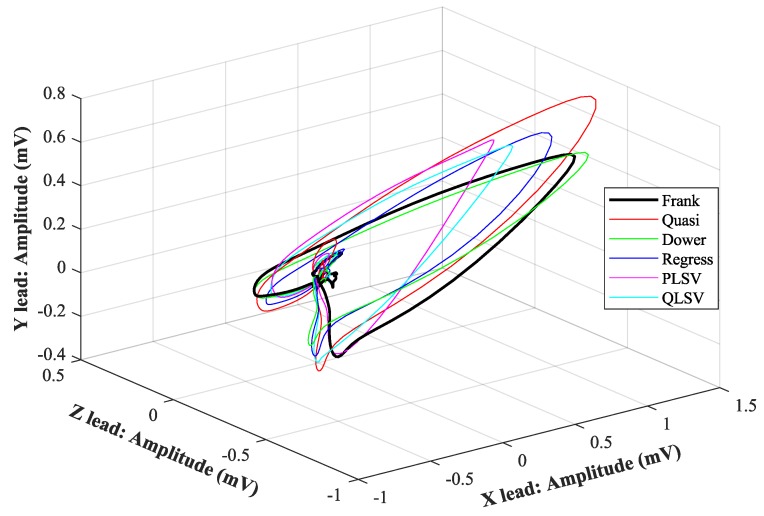
Comparison of transformation methods in 3-D, random patient 2.

**Figure 11:**
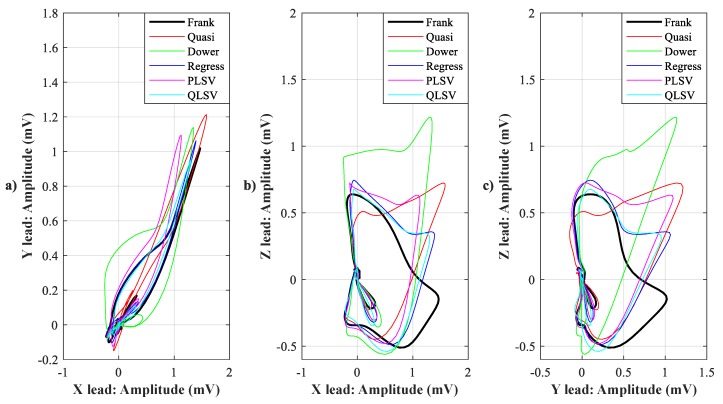
Comparison of transformation methods in 2-D, random patient 3. (**a**) X lead to Y lead, (**b**) X lead to Z lead, and (**c**) Y lead to Z lead.

**Figure 12:**
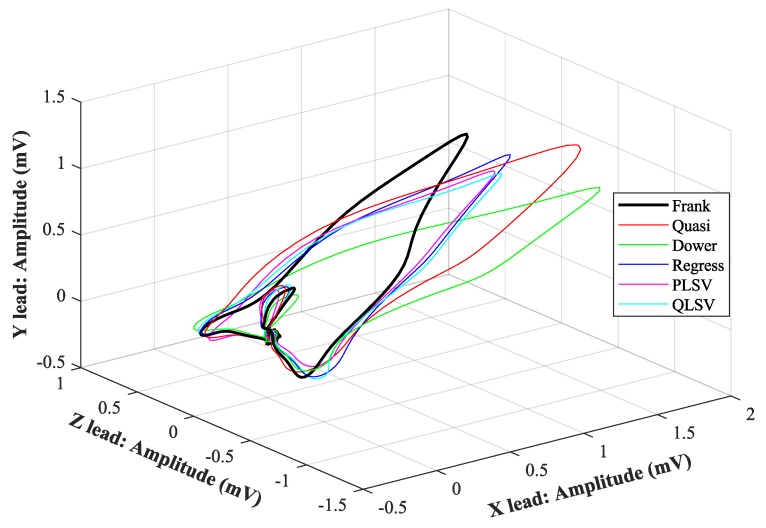
Comparison of transformation methods in 3-D, random patient 3.

**Figure 13:**
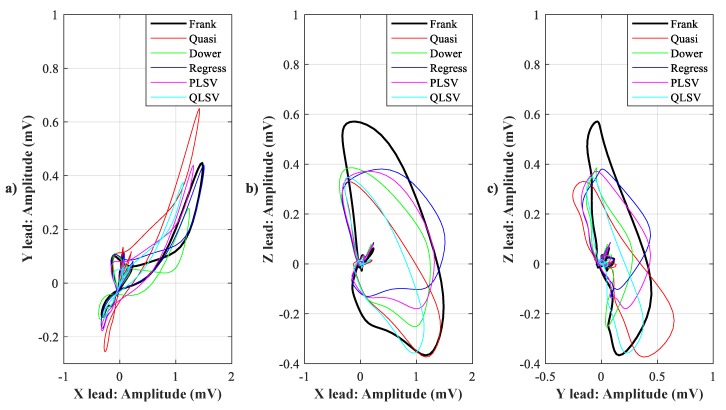
Comparison of transformation methods in 2-D, random patient 4. (**a**) X lead to Y lead, (**b**) X lead to Z lead, and (**c**) Y lead to Z lead.

**Figure 14:**
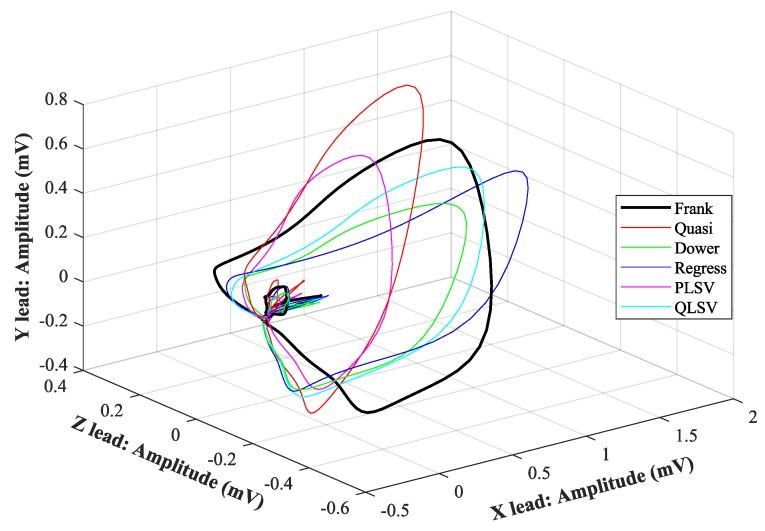
Comparison of transformation methods in 3-D, random patient 4.

In Figure 7, Figure 8, Figure 9, Figure 10, Figure 11, Figure 12, Figure 13 and Figure 14, you can see 2-D and 3-D comparisons of tested tranformations. For visual comparison, we chose four random recordings out of the whole tested data set. 2-D figures were selected, as they are ideal for comparisons to the reference Frank’s orthogonal lead system. According to the MSE and R parameters, Kors regression transformation provided the best results, which also corresponds to the mentioned figures.

Kors regression transformation has advantages over Frank’s orthogonal lead system. It is much easier to identify myocardial infarction [41,42,43,44,45,46], hypertrophy [47,48,49], and ischaemic heart disease [50,51] from the VCG compared to the ECG. However it is important to properly estimate the VCG from the ECG. Transformations need a lower number of required leads in comparison to Frank’s orthogonal lead system combined with the conventional ECG system. To better diagnose heart diseases, doctors often require both signals at once. In the case of a successful extraction of the VCG from the ECG, it is possible to identify multiple symptoms, such as the QRS-T angle, QRS-T spatial angle, heart axis slope, and QRS loop vector size [52]. A high accuracy of Kors regression transformation could lead to a complete deprecation of Frank’s orthogonal lead system, which also provides higher comfort for patients.

Apart from the presented solutions, a number of different VCG transformations were tested by other teams, with varying results. Other teams carried out experiments based on affine transform, PCA, or transformation methods based on artificial neural networks that offer even higher accuracy [25,26,27]. These transformations will be a basis for our future research.

VCG systems based on transformations are much cheaper to conventional Frank’s orthogonal lead system, since it does not need a dedicated hardware platform. Newly graduated doctors could potentially choose this system for their clinical practice, since it has significantly lower initial costs.

## 6. Conclusions

The accuracy of the ECG transformation to the VCG is very important for possibly determining symptoms of hypertrophy, ischaemic heart disease, etc. A total of five different transformation methods, which were tested on PTB database recordings, were selected for the testing. The results of the transformations were compared with the original Frank’s leads measured on the basis of the MSE and R. From the results, the medians of the individual results were determined and statistical analysis was performed with emphasis on Kors regression transformation in relation to other transformation methods. The results were determined separately for each lead. A nonparametric statistical test was used to calculate the *p*-values by means of which the significance of the difference of values between the transformations was determined.

During testing, Kors regression transformation was statistically the most accurate for X and Y leads compared to other transformation methods. The smallest accuracy of transformation was provided by Kors quasi-orthogonal transformation. The difference between Kors regression transformation and the PLSV and QLSV methods was not statistically significant for Z lead. Overall, the results showed that Kors regression transformation was the most accurate of the methods tested and could be significant for transforming the ECG into the VCG for automatic computer analysis of ECG recordings based on VCG symptoms. Evaluating the accuracy of transformation methods for patients suffering from some heart disease and comparing the effects of transformations on pathological recordings could be the subject of the next work. Furthermore, newer types of transformation methods could be added. In the future, ECG transformations into VCG could be used in clinical practice, which could facilitate diagnosing heart diseases.

2-D/3-D graphs (Figure 7, Figure 8, Figure 9 and Figure 10) were reconstructed after processing tested transformations via statistical analysis. 2-D/3-D graphs consisting of VCG loops are very important in clinical practice, as they are provide more information than the ECG and therefore help doctors identify heart diseases. Comparisons of visual signals correlates with statistical analysis. Kors regression transformation tend to be the most similar to Frank’s orthogonal lead system, so its safe to say that it could be used for morphological analysis of 2-D/3-D graphs (for example pathological phenomena).

## Figures and Tables

**Table 1:** Transformation matrix coefficients for inverse Dower transformation.

Lead	X	Y	Z
**V1**	−0.172	0.057	−0.229
**V2**	−0.074	−0.019	−0.310
**V3**	0.122	−0.106	−0.246
**V4**	0.231	−0.022	−0.063
**V5**	0.239	0.041	0.055
**V6**	0.194	0.048	0.108
**I**	0.156	−0.227	0.022
**II**	−0.010	0.887	0.102

**Table 2:** Coefficients for Kors regression transformation.

Lead	X	Y	Z
**V1**	−0.130	0.060	−0.430
**V2**	0.050	−0.020	−0.060
**V3**	−0.010	−0.050	−0.140
**V4**	0.140	0.060	−0.200
**V5**	0.060	−0.170	−0.110
**V6**	0.540	0.130	0.310
**I**	0.380	−0.070	0.110
**II**	−0.070	0.930	−0.230

**Table 3:** Coefficients for the P wave (PLSV) method.

Lead	X	Y	Z
**V1**	0.147	0.023	0.184
**V2**	0.058	0.085	0.163
**V3**	0.037	0.003	0.190
**V4**	0.139	0.033	0.119
**V5**	0.232	0.060	0.023
**V6**	0.226	0.104	0.043
**I**	0.199	0.146	0.085
**II**	0.018	0.503	0.130

**Table 4:** Coefficients for the QRS complex (QLSV) method.

Lead	X	Y	Z
**V1**	−0.266	0.088	−0.319
**V2**	0.027	−0.088	−0.198
**V3**	0.065	0.003	−0.167
**V4**	0.131	0.042	−0.099
**V5**	0.203	0.047	−0.009
**V6**	0.22	0.067	0.06
**I**	0.37	−0.131	0.184
**II**	−0.154	0.717	−0.114

**Table 5:** Table of the medians of the MSE results for individual transformation methods and individual leads and the resulting *p*-values between the medians of Kors regression transformation and the medians of other transformation methods for individual leads.

Mean Squared Error	X	Y	Z
**Regress Median**	2.2024 · 10${}^{-4}$	3.8445 · 10${}^{-4}$	1.9622 · 10${}^{-3}$
**Quasi Median**	1.5799 · 10${}^{-3}$	1.9417 · 10${}^{-3}$	3.8956 · 10${}^{-3}$
**Dower Median**	1.4880 · 10${}^{-3}$	8.7119 · 10${}^{-4}$	6.5558 · 10${}^{-3}$
**PLSV Median**	1.1881 · 10${}^{-3}$	7.2627 · 10${}^{-4}$	2.3116 · 10${}^{-3}$
**QLSV Median**	1.1887 · 10${}^{-3}$	1.0313 · 10${}^{-3}$	2.5286 · 10${}^{-3}$
***p*-value (Regress × Quasi)**	2.4982 · 10${}^{-11}$	3.5755 · 10${}^{-12}$	1.9268 · 10${}^{-4}$
***p*-value (Regress × Dower)**	1.2882 · 10${}^{-9}$	5.7956 · 10${}^{-5}$	1.1888 · 10${}^{-7}$
***p*-value (Regress × PLSV)**	2.7719 · 10${}^{-8}$	3.6261 · 10${}^{-4}$	0.2878
***p*-value (Regress × QLSV)**	4.9807 · 10${}^{-9}$	3.2827 · 10${}^{-7}$	0.3576

**Table 6:** Table of the medians of the R results for individual transformation methods and individual leads and the resulting *p*-valuess between the medians of Kors regression transformation and the medians of other transformation methods for the individual leads.

Correlation Coefficient	X	Y	Z
**Regress Median**	0.998397	0.994343	0.981839
**Quasi Median**	0.986784	0.986223	0.948075
**Dower Median**	0.990345	0.982841	0.944712
**PLSV Median**	0.990350	0.982036	0.966346
**QLSV Median**	0.994695	0.976752	0.971982
***p*-values (Regress × Quasi)**	2.5482 · 10${}^{-9}$	3.2827 · 10${}^{-4}$	3.0635 · 10${}^{-3}$
***p*-values (Regress × Dower)**	6.0021 · 10${}^{-8}$	2.4887 · 10${}^{-4}$	3.6261 · 10${}^{-4}$
***p*-values (Regress × PLSV)**	2.1785 · 10${}^{-6}$	9.7206 · 10${}^{-5}$	0.2485
***p*-values (Regress × QLSV)**	7.1535 · 10${}^{-6}$	1.4784 · 10${}^{-6}$	0.1612
